# A Criticism of the New Nomenclature

**Published:** 1914-04-04

**Authors:** 


### DEFINITIONS OF MENTAL DEFICIENCY.

### A Criticism of the New Nomenclature.

The subject of heredity in relation to mental disorder is undoubtedly of widespread interest at the present time, when the outcry for social legislation has led to the Mental Deficiency Act, which came into force on April 1. The difficulties of the subject are great; many theories have been advanced, but the evidence to support them is incomplete. Even the question of who ought to be the authority on the subject gives rise to argument. Medical men say that they are best fitted; statisticians believe that it is a matter for them; and one writer, who is presumably a biologist, states that problems of the race are biological questions of variation, heredity and evolution, and apparently holds that biologists are the elect. Meanwhile the majority of thinking people, being principally interested in results, pray that all will work harmoniously together to obtain them; and the general public greedily absorb any sensational piece of news on the subject which appears in the daily press.

In two late numbers of "Questions of the Day and of the Fray" (a series of pamphlets published by the workers in the Galton Eugenics Laboratory of the University of London) there is a great deal that is of interest. These two numbers are entitled "Mendelism and the Problem of Mental Defect," Part I. being "A Criticism of Recent American Work," by David Heron, D.Sc., and Part II. "On the Continuity of Mental Defect," by Professor Karl Pearson and Gustav A. Jaederholm. The first of these is a destructive criticism of some recent American publications on "Heredity and Eugenics," by Dr. C. B. Davenport and others, who state that mental defect is a unit character and a Mendelian recessive. This and other statements of deductions made by Dr. Davenport from the contents of these publications were made at the International Eugenics Congress and appeared in the English daily press. They were so didactic as to require the strongest critical examination before being accepted, and in the first of these papers this strong criticism is brought to bear.

It shows how easily workers who are obsessed by one particular theory can make facts fit that theory, and can ignore those which do not fit it. It further points out clearly how much the personal equation of the observer enters into the results, and how the worker can gain the support and approbation of those who are properly biassed in the same direction. It is impossible to quote extracts, but the whole should be read by everyone interested in the subject: the lessons to be learnt from it are good.

Part II. gives the results of the examination of 300 mentally defective and 250 normal children in the schools of Stockholm. A part of the pamphlet is a statistical study of a highly technical character, which requires special training to follow, but the information about the schools and the results obtained is worth reading. At the outset the writers remark that one of the great difficulties attending legislation for the mentally defective lies in the need of an adequate definition of "mental defect." Anyone who reads this pamphlet cannot fail to be impressed with this need. The writers apparently judge mental defect entirely by standard of intelligence as shown by tests of the Binet-Simon type. To them degree of intelligence appears to be the only standard of mental normality. They suggest that "social inefficients" would be a better term for those who, though possessing intelligence equal to many normals, yet lack power of self-control and attention, are faulty in habits of cleanliness, are immoral, have fits, etc.; and deplore the fact that the mentally deficient are now drafted to the special schools by the combined personal equation of the teachers, school nurses, and doctors. The two following quotations will, we think, make this point clear:—

"As far as mental state is concerned, all we can wisely do is to take some test like the Binet-Simon—when it has been finally established or modified by wider experience—and say, when a child lacks mentally four, five, or whatever limit of years be chosen of its physical age, then this child shall have special provision made for it. This is all we can do for the mentally deficient; for the socially inefficient a mental test cannot cover the ground. . . ."

And again: ". . . This 'hotch-potch' of physical defect, feeble-mindedness, and social inefficiency, which has been, and will now be, still further segregated on the basis of 'school inefficiency,' is what passes as a 'mental defect.' . . ."

If we assume for the moment that Professor Pearson's definition of mental deficiency is the correct one, we can agree with what he says. But is the definition the right one? Does mental deficiency mean merely "a certain amount below average intelligence"? We think that this is a very narrow definition, and not the one usually accepted—certainly not the one implied in the Mental Deficiency Act, 1913. To say that intelligence, as judged by the Binet-Simon test, and mind are synonymous terms seems to be a little unusual. The word mind is generally taken as connoting more than intelligence. In judging mental defect it is usual, we think, to examine other things besides intelligence, amongst them the very ones he mentions—viz. power of self-control and attention, moral sense, habits of cleanliness, etc., taking into account the upbringing of the individual. The more usual term for children who are merely below the average of intelligence is "backward." Professor Pearson seems to be forcing a new definition of mental deficiency on us, presumably because there are signs that it will be possible in the future to express intelligence in exact mathematical terms. If this definition is adopted, we think it may lead to much confusion. The mental state of adults is not judged alone by intelligence, as shown by tests of the Binet-Simon type; and, unless changes are made in the nomenclature as applied to adults as well, mind would mean something different in an adult and a child.

That merely backward children should receive special attention, but not be classed with mentally deficients, most people will agree; but, with the usual definition of mental deficiency, the only test at present possible appears to be "medico-pedagogic opinion."

